# Perception of interrupted speech and text: Listener and modality factorsa)

**DOI:** 10.1121/10.0011571

**Published:** 2022-06-03

**Authors:** Daniel Fogerty, Judy R. Dubno, Valeriy Shafiro

**Affiliations:** 1 Speech and Hearing Science, University of Illinois Urbana-Champaign, 901 South Sixth Street, M/C 482, Champaign, Illinois 61820, USA; 2 Otolaryngology-Head and Neck Surgery, Medical University of South Carolina, 135 Rutledge Avenue, MSC 550, Charleston, South Carolina 29425, USA; 3 Communication Disorders and Sciences, Rush University Medical Center, 1015 Armour Academic Building, 600 S. Paulina Street, Chicago, Illinois 60612, USA dfogerty@illinois.edu, dubnojr@musc.edu, Valeriy_Shafiro@rush.edu

## Abstract

Interrupted speech and text are used to measure processes of linguistic closure that are important for recognition under adverse backgrounds. The present study compared recognition of speech and text that had been periodically interrupted with matched amounts of silence or white space, respectively. Recognition thresholds were obtained for younger and older adults with normal or simulated/impaired hearing and correlated with recognition of speech-in-babble. Results demonstrate domain-general, age-related processes in linguistic closure affecting high context sentences and domain-specific, hearing-related processes in speech recognition affecting low context sentences. Text recognition captures domain-general linguistic processes in speech recognition susceptible to age-related effects.

## 1. Introduction

In noisy listening environments, speech signals reaching the listener are often incomplete and contain highly fragmented audible information about underlying linguistic elements. Nevertheless, listeners can usually maintain high perceptual accuracy even when substantial portions of the signal are masked or deleted (Miller and Licklider, 1950). Similar effects have been also observed in the perception of fragmented text, known as linguistic closure (Besser *et al.*, 2013). In an interrupted text task, a visual analogue to the interrupted speech paradigm developed by Miller and Licklider (1950), portions of a text string are periodically replaced by vertical filled bars or white space (George *et al.*, 2007; Zekveld *et al.*, 2007) with the width of the bars controlling the proportion of text shown. This interrupted text task is somewhat analogous to Shannon's (1951) letter-guessing procedure where participants had to guess letters in a passage of text based on preceding letters. It was later adapted by van Rooij and Plomp (1991) to demonstrate that predictability of test materials can have a measurable effect on speech recognition in younger and older adults.

Indeed, both speech and text provide modality-specific representations of the underlying modality-general linguistic structure. Their processing may thus be influenced by an overlapping set of modality-general perceptual, cognitive, and linguistic factors (Humes *et al.*, 2013), which may in turn contribute to the variance observed in perception of fragmented speech (Saija *et al.*, 2014; Shafiro *et al.*, 2016). Associations between interrupted text and masked or interrupted speech have been observed in some but not all studies (for a review, see Besser *et al.*, 2013). Generally, the strength of associations appears to be influenced by listener and stimulus specific factors, including working memory capacity, age, hearing loss, speech clarity, and the proportion and rate of the signal preserved after interruptions. Significant correlations between interrupted text and speech appear more likely in older and in hearing impaired adults than younger and normal hearing listeners and in sentences rather than words (Zekveld *et al.*, 2018).

The relationship between interrupted text and speech could also be affected by the nature of perceptual units that are preserved after interruption or masking. Because linguistic processing operates on different time scales (Giraud and Poeppel, 2012; Chait *et al.*, 2015), and involves elements of different length, such as words, syllables, and phonemes, total proportion of signal preserved may provide a skewed sense of the linguistic information available in the signal. For example, a text string or a spoken utterance can be interrupted at different rates, leaving either word-size or phoneme/grapheme-size gaps, even as the total proportion of speech or text remains the same. To control both parameters of interruption rate and proportion of speech/text preserved more systematically, Shafiro *et al.* (2018) proposed a method to convert the duration of silent intervals used to interrupt speech to the width of white space bars used to interrupt text. In this way, the preserved proportion was held constant while information for speech and text was distributed across the message at different rates, from large interruptions preserving whole words and phrases to small interruptions preserving phoneme fragments. Results indicated that, in younger normal hearing adults, recognition accuracy varied as a function of the number of words preserved within an interruption and the number of interruptions per word. These two parameters had a similar influence on recognition of both interrupted speech and text, suggesting a modality-general effect whereby the distribution of perceptual information affects access to the underlying linguistic elements. However, younger and older adults differ in recognition errors at the phoneme and word level (Smith and Fogerty, 2021), indicating likely age effects.

In the present study, we investigated the effects of age and hearing loss on the perception of interrupted speech and text as a function of the proportion of the signal preserved. Importantly, whereas previous studies have investigated associations between interrupted text and masked or interrupted speech (see Besser *et al.*, 2013), they have not controlled for the distribution of preserved signal information by matching interruption rate. In this study, we focused on an interruption rate of 2 Hz, which represents the rate at which speech and text recognition is most disrupted (Shafiro *et al.*, 2018), while speech recognition is most affected by advancing age (Saija *et al.*, 2014; Shafiro *et al.*, 2016). Therefore, the purpose of the present study was to investigate the role of linguistic closure in the perception of speech and text, interrupted to match the amount of preserved and deleted signal information, in a heterogeneous sample of younger and older adults with normal or impaired hearing. A secondary goal was to assess the mediating effect of age and hearing on the relationship of interrupted speech and text to the perception of speech which was masked by a different type of common masker—multitalker babble.

## 2. Methods

### 2.1 Participants

Four groups of adults participated in this experiment. Twenty younger adults with normal hearing (YNH; 19–28 years, mean [M] = 23 years), 20 older adults with normal hearing (ONH; 60–74 mean [M], M = 67 years), 21 older adults with hearing impairment (OHI; 62–84 years, M = 72 years) and 23 younger adults with normal hearing who listened to spectrally shaped speech and noise to simulate hearing loss (YSH; 18–24 mean [M], M = 20 years). YNH and YSH adults had pure-tone thresholds ≤20 dB HL at octave frequencies from 0.25–8 kHz (ANSI, 2004). ONH and OHI adults had thresholds ≤25 dB HL or ≤55 dB HL at 4 kHz and below, respectively. Four-frequency pure-tone average (PTA4–500, 1000, 2000, and 4000 Hz) was used as a summary variable (YNH: M = −1.2 dB HL; SD = 3.6; YSH: M = 1.5 dB HL, SD = 3.3; ONH: M = 11.3 dB HL, SD = 3.8; OHI: M = 29.8 dB HL, SD = 8.4). All participants were native speakers of American English and all older adults obtained a score of 25 or better on the Mini Mental State Exam (MMSE) (Folstein *et al.*, 1975). According to the World Health Organization (2019), Snellen Visual Acuity Test scores for corrected vision indicated that 38 older adults had no visual impairment and three had mild visual impairment (older range = 20/15 to 20/50, mean = 20/32). A subset of 28 younger adults also completed this screen of visual acuity (younger range = 20/15 to 20/40, mean = 20/22). Younger and older adults were recruited as part of a larger study, with initial reports detailing group differences in speech-modulated noise (Fogerty *et al.*, 2020), as well as in microstructural analyses of errors as a function of noise modulation depth (Fogerty *et al.*, 2021) and speech misperceptions in multitalker babble (Vickery *et al.*, 2022).

### 2.2 Speech and text interruption

**Fig. 1.**
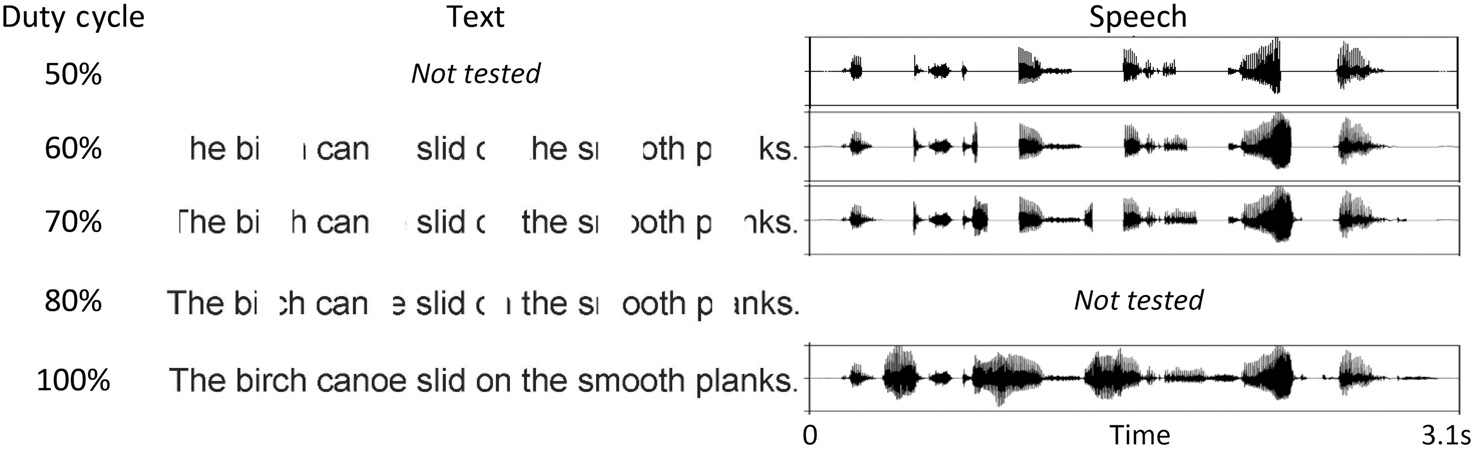
Example sentence for 2 Hz interrupted speech and text preserved at different duty cycles.

Experimental stimuli were sentences selected from the Harvard/IEEE corpus (IEEE, 1969) with five key words per sentence (example: The *birch canoe slid* on the *smooth planks*). Figure 1 displays example stimuli. Speech and text stimuli were matched for presentation time, interruption rate (2 Hz), and duty cycle (i.e., proportion of signal preserved) across each sentence as in Shafiro *et al.*, (2018) to approximate interruption time in milliseconds in space taken in pixels. That is, auditory and visual versions of the same sentence contained the same number of interruptions (see Shafiro *et al.*, 2018 for additional details). Silence and white space were used for interrupting speech and text, respectively. Speech interruptions were conducted at 50%, 60%, and 70% duty cycles, whereas text interruptions were based on 60%, 70%, and 80% duty cycles. These duty cycles were selected to avoid floor and ceiling performance. Speech and text recognition were also measured at 100% duty cycle (i.e., uninterrupted) to obtain a measure of maximum performance. The starting phase (or pixel location) of the interruption cycle was randomized for each sentence. For auditory speech presentations, interruption windows used a 2 ms raised cosine on/off ramp.

### 2.3 Speech processing

Stimuli were scaled to 70 dB SPL, prior to interruption, and then spectrally shaped, by increasing gain in 1/3 octave bands, according to the individual thresholds of the YNH, ONH, or OHI listeners to ensure at least 15 dB sensation level (SL) through at least 4 kHz. In practice, this resulted in minimal changes in the spectrum for YNH and ONH listeners (i.e., 1/3 octave band SLs already exceeded 15 dB and required no further gain) with an average speech level maintained at 70 dB SPL. The average speech level following OHI spectral shaping was 82 dB SPL. The YSH group listened to spectrally shaped speech that was identical to the shaped speech provided to individual OHI participants to which they were paired. In addition, speech was presented in the presence of a threshold matching noise with a spectrum that matched the hearing thresholds of the paired OHI listener in order to equate sensation levels between OHI and YSH listeners. Previous analysis with these same listeners has indicated that the combination of threshold matching noise and spectral shaping provided to the YSH group is sufficient to model the benefit OHI listeners obtain from glimpsing speech in speech-modulated compared to unmodulated noise (Fogerty *et al.*, 2020). Finally, speech stimuli were passed through a low-pass, linear phase, finite-impulse-response, 128th-order filter with a cutoff of 5.623 kHz in order to minimize the effect of high frequency hearing loss in the OHI group, for which full audibility could not be obtained.

### 2.4 Speech-in-babble testing

Speech-in-babble testing was conducted to examine associations with a more ecologically appropriate listening condition. The Revised Speech in Noise test (R-SPIN; Bilger *et al.*, 1984) was presented in 12-talker babble at 0 dB signal-to-noise ratio (SNR). One list (a total of 50 sentences) was presented, with sentences divided in half according to low and high context sentence contexts. Participants were required to repeat the final word, which was transcribed live and recorded for offline analysis. Performance on this task was previously reported (Vickery *et al.*, 2022) and included here as an outcome measure for prediction by interrupted speech and text.

### 2.5 Procedures

Participants completed testing in a sound-attenuating booth. Speech stimuli were presented at a sampling rate of 48.828 kHz *via* one of a pair of Sennheiser (Wedemark, Germany) HDA 200 headphones following a TDT (Alachua, FL) System III digital-to-analog processor (RP2/RX6) and headphone buffer (HB7/HB5). Presentation was monaural to the right ear, unless target sensation levels were better obtained using the left ear (3 ONH, 14 OHI). Text stimuli were displayed as full sentences for each condition, centered on an ELO (Milpitas, CA) touchscreen monitor in a visual text window (1600 × 200 pixels).

A block of four familiarization trials was provided prior to testing that exposed participants to the range of duty cycles tested. Ten sentences were presented per duty cycle condition, with a total of 40 sentences for each modality. Testing proceeded from the smallest duty cycle (i.e., 50% or 60% for speech or text, respectively) to the greatest (i.e., 100%) to minimize perceptual learning across trials.

Open-set responses were live scored. Participants were encouraged to guess. No feedback was provided. A response was scored as correct if the participant repeated each key word exactly (e.g., without missing or extra phonemes). Data across the duty cycle conditions for each participant were fit to individual logistic functions and the 50% recognition threshold for speech (SRT50) and text (TRT50) were calculated. These values reflect the duty cycle (i.e., amount of preservation) required to obtain 50% correct speech/text recognition.

## 3. Results

### 3.1 Comparisons between groups

All participants had adequate hearing and visual acuity to perceive the speech and text stimuli, as indicated by obtaining at least 90% correct for uninterrupted speech and text (i.e.,100% duty cycle), with the exception of one OHI who obtained 86% for uninterrupted speech.

**Fig. 2.**
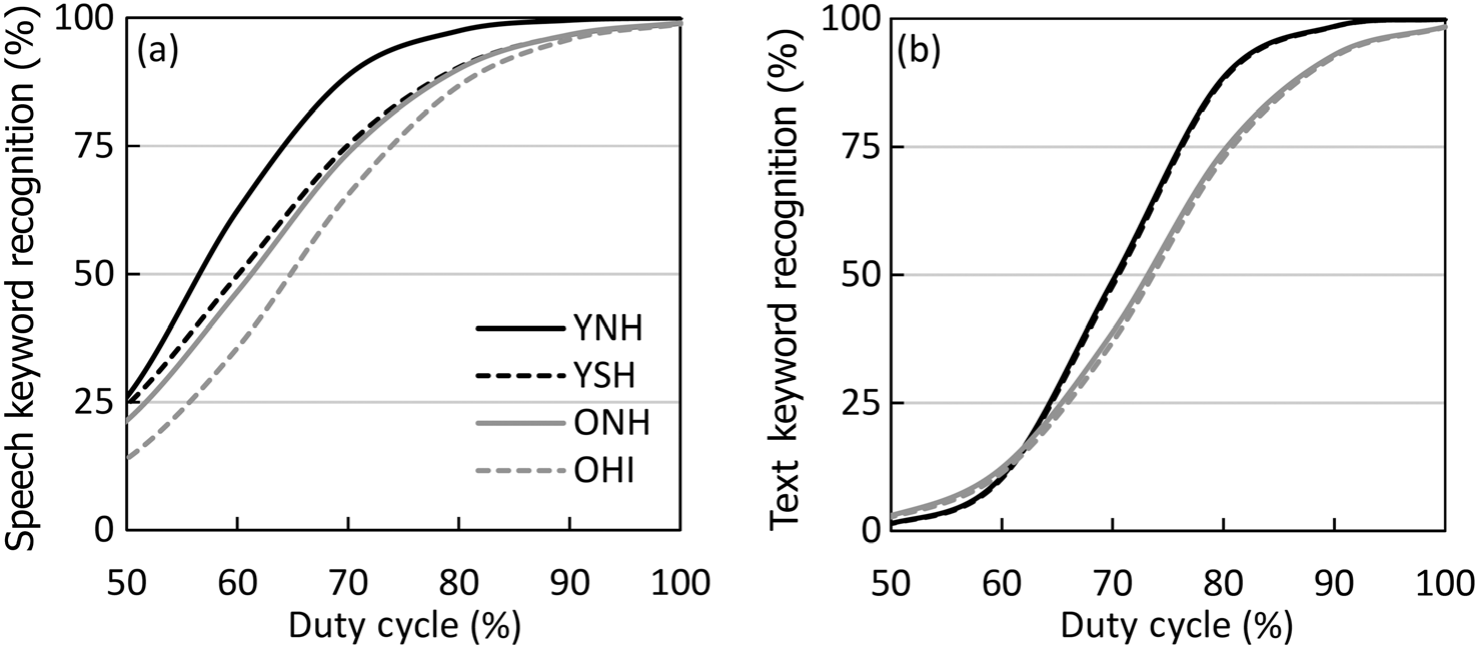
Logistic functions fit to group performance across duty cycles [i.e., percent of (a) speech or (b) text preserved]. Thresholds were defined as the duty cycle required to obtain 50% correct recognition.

As a summary of performance, logistic functions fit to the group average for interrupted speech and text are displayed in Fig. 2. As can be observed from the functions, group differences associated with age and hearing loss are apparent when listening to interrupted speech [Fig. 2(a)], but only age effects are observed for interrupted text [Fig. 2(b)]. These differences are most pronounced at higher levels of text preservation. Furthermore, functions for text occur within a narrower range compared to speech (steeper functions), indicating the recognition of interrupted text declines more rapidly with decreases in duty cycle.

As can be observed from Fig. 2, higher recognition rates (i.e., better performance) were obtained for interrupted speech compared to interrupted text. A mixed model analysis of variance (ANOVA) investigated the effects of modality and group. A significant main effect of modality, F(1,80) = 647.4, p < 0.001, η_p_^2^ = 0.89, was found, confirming better recognition for interrupted speech with less perceptual information (i.e., smaller duty cycles). Also, observed was a main effect of group, F(3, 80) = 14.6, p < 0.001, η_p_^2^ = 0.35, with a significant interaction, F(3,80) = 7.4, p < 0.001, η_p_^2^ = 0.22. Regarding the group effects, *post hoc* paired t-tests between groups demonstrated an effect of simulated and actual hearing loss for SRT50 [YNH vs YSH: t(41) = −3.3, p = 0.002; ONH vs OHI: t(39) = −2.1, p = 0.046]. However, the comparison among older adults did not reach significance following Bonferroni correction for multiple comparisons. As expected, no effect of simulated or actual hearing loss occurred for TRT50 (YNH vs YSH: p = 0.458; ONH vs OHI: p = 0.541). An effect of age was observed for both SRT50 [YNH vs ONH: t(38) = −4.0, p < 0.001; YSH vs OHI: t(42) = −3.7, p = 0.001] and TRT50 [YNH vs ONH: t(38) = −3.0, p = 0.005; YSH vs OHI: t(42) = −2.6, p = 0.012]. Thus, these results demonstrated that the group effect for SRT50 was mediated by both age and reduced hearing, whereas the effect of TRT50 was mediated by age-related factors alone.

### 3.2 Correlational analysis

**Fig. 3.**
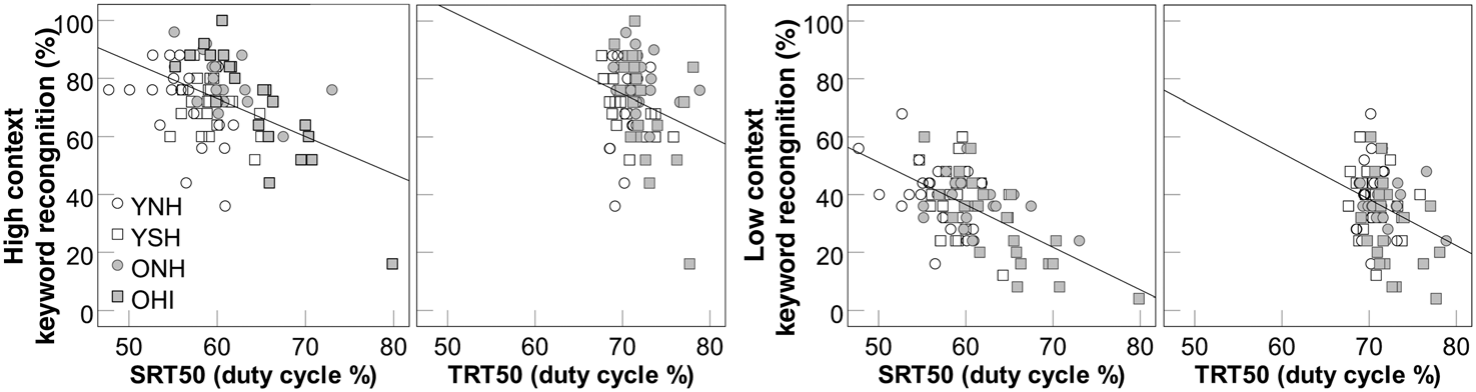
Scatterplots for speech (SRT50) and text (TRT50) recognition thresholds at 50% correct across the four groups in relation to R-SPIN scores for high context and low context sentences in multitalker babble.

All 84 participants were entered into Spearman rank order (r_s_) partial correlation analyses, controlling for age and PTA4. Results demonstrated a weak but nonsignificant positive correlation between speech and text thresholds (r_s_ = 0.19, p = 0.09). To assess the generalization of interrupted speech and text measures to more realistic speech-in-babble recognition, partial correlations were also tested with R-SPIN sentence recognition. As shown in Fig. 3, SRT50 was significantly negatively correlated with high (r_s_ = −0.50, p < 0.001) and low (r_s_ = −0.43, p < 0.001) context sentences, accounting for around 30% of the variance of speech recognition in multitalker babble. TRT50 was weakly negatively correlated with high (r_s_ = −0.26, p = 0.02) and non-significantly with low (r_s_ = −0.14, p = 0.226) context sentences. Higher speech and text recognition thresholds, which indicated that participants required greater preservation of the stimulus, were associated with poorer speech-in-babble performance. Using Zou (2007) confidence intervals (CI) from the cocor package in R (Diedenhofen and Musch, 2015), SRT50 correlation coefficients were significantly larger than TRT50 coefficients for high (95% CI: −0.4899, −0.0513) and low (95% CI: −0.5341, −0.0828) context sentences.

High and low context sentences were significantly correlated (r_s_ = 0.56, p < 0.001), reflecting shared variance in indexing general speech-in-babble recognition abilities. As TRT50 was only significantly associated with high context sentences, low content sentence recognition was added as a control variable along with age and PTA4. SRT50 (r_s_ = −0.34, p = 0.002) and TRT50 (r_s_ = −0.22, p = 0.049) were associated with the unique variance in performance on the high context sentences. In this case, the correlation coefficients for SRT50 and TRT50 on the unique variance in high context sentences were not significantly different (95% CI: −0.3766, 0.1005).

## 4. Discussion

The present findings examined recognition of similarly interrupted speech and text to determine contributions of modality-general and modality-specific factors. The interruption parameters for speech and text were chosen to be analogous and provide matching signal representation in both modalities. Logistic functions were used to describe recognition performance and compare the proportion of preserved speech and text needed to reach an estimated 50% correct threshold. Results indicate only a weak association between interrupted speech and text, when controlling for age and hearing loss. This suggests an overall low modality-general effect of linguistic closure, even though the visual and auditory signal information is maximally matched between both modalities. Therefore, similarity between underlying linguistic elements achieved by matching interruption parameters for text and speech does not fully account for the domain-specific processing in each modality.

The correlation analysis with R-SPIN sentences further highlight the role of domain-general and domain-specific processing of interrupted sentences. Specifically, the present results reveal significant correlations of both interrupted text and speech with high context masked R-SPIN sentences that have semantic cues that aid key word identification. Indeed, when controlling for general speech-in-babble abilities, SRT50 and TRT50 are similar in explaining linguistic closure abilities. In contrast, interrupted speech but not interrupted text scores also explain additional variance on low context masked R-SPIN sentences for which key word identification is much less constrained by contextual cues. This pattern of results suggests that the modality-general effect of linguistic closure facilitates key word recognition in semantically predictable contexts for both text and speech, while modality-specific factors of speech-in-babble processing play a greater role when contextual cues are limited, such as the case with low context R-SPIN sentences. Consistent with the purpose and design of the TRT task (Zekveld *et al.*, 2007), TRT50 specifically assesses linguistic closure related to higher order modality-general language processing, but is independent of domain-specific aspects of speech-in-babble processing related to auditory perception.

The role of modality-general and modality-specific factors can be further examined in the effects of age and hearing loss. The comparison of performance across the four groups of the present study, which differ in participant age and hearing abilities, indicates that both age and hearing loss influence the perception of interrupted speech [Fig. 2(a)]. However, only age had an effect on the perception of interrupted text, with older adults requiring a greater proportion of text to be visible to reach the same level of performance as younger adults [Fig. 2(b)]. Thus, the modality-specific factor, hearing loss, affects perception of interrupted speech but not text, while a modality-general factor, age, affects sentence recognition in both modalities. Furthermore, age is known to be associated with other modality-general factors such as working memory capacity and lexical access speed, which could be the underlying contributing factors to performance for both interrupted text and speech (Zekveld *et al.*, 2018; Besser *et al.*, 2013; Humes *et al.*, 2013).

Overall, the present findings indicate that both age and hearing loss have a negative influence on the perception of interrupted speech, while only age affects perception of interrupted text. Stronger correlations with sentences in babble with interrupted speech than text, while controlling for age and hearing loss, were likely due to these modality-specific factors. Although perception of both interrupted speech and text involves modality-general linguistic closure, which can compensate for missing linguistic elements in the sensory signal, other factors such as age, hearing ability, and signal quality may affect perceptual processing, leading to modality-specific differences in perceptual outcomes. Overall, the study findings indicate that the perception of interrupted speech is a stronger predictor than interrupted text for the recognition of speech in babble.
